# The complete chloroplast genome sequence of *Litsea garrettii*

**DOI:** 10.1080/23802359.2020.1726225

**Published:** 2020-02-11

**Authors:** Qiong Qiu, Dejun Yang, Linhong Xu, Yumei Xu, Yi Wang

**Affiliations:** a Institute of tropical forestry, Yunnan Academy of Forestry, Puwen, Yunnan, People’s Republic of China;; b The Key Laboratory of Rare and Endangered Forest Plants of State Forestry Administration, Kunming, Yunnan, People’s Republic of China

**Keywords:** *Litsea garrettii*, chloroplast, Illumina sequencing, phylogenetic analysis

## Abstract

The first complete chloroplast genome (cpDNA) sequence of *Litsea garrettii* was determined from Illumina HiSeq pair-end sequencing data in this study. The cpDNA is 154,011 bp in length, contains a large single-copy region (LSC) of 93,697 bp and a small single-copy region (SSC) of 18,826 bp, which were separated by a pair of inverted repeat (IR) regions of 20,744 bp. The genome contains 127 genes, including 82 protein-coding genes, 8 ribosomal RNA genes, and 36 transfer RNA genes. Further phylogenomic analysis showed that *L. garrettii* and *Parasassafras confertiflorum* clustered in a clade in Lauraceae family.

*Litsea garrettii* is the species of the genus *Litsea* within the family Lauraceae, distributed in south Yunnan, China, Thailand, and Myanmar (Li et al. 2008). *Litsea garrettii* is a Chinese traditional medicine used for treatment of swelling caused by injury and snake biting (Yan et al. 2018). The extract of *L. garrettii* had potential preventive effects against inflammation and oxidative stress-related diseases (Li et al. 2018). The prototypic sesquiterpenes from *L. garrettii* demonstrated anti-HIV activity (Guan et al. 2016). Therefore, *L. garrettii* has huge medicinal value. However, there has been no genomic studies on *L. garrettii.*

Herein, we reported and characterized the complete *L. garrettii* plastid genome. The GenBank accession number is MN698967. One *L. garrettii* individual (specimen number: 20180329) was collected from Cangyuan, Yunnan Province of China (23°18′59″ N, 99°07′32″ E). The specimen is stored at Yunnan Academy of Forestry Herbarium, Kunming, China and the accession number is YAFH0012765. DNA was extracted from its fresh leaves using DNA Plantzol Reagent (Invitrogen, Carlsbad, CA, USA).

Paired-end reads were sequenced by using Illumina HiSeq system (Illumina, San Diego, CA, USA). In total, about 21.6 million high-quality clean reads were generated with adaptors trimmed. Aligning, assembly, and annotation were conducted by CLC de novo assembler (CLC Bio, Aarhus, Denmark), BLAST, GeSeq (Tillich et al. 2017), and GENEIOUS v 11.0.5 (Biomatters Ltd, Auckland, New Zealand). To confirm the phylogenetic position of *L. garrettii*, other 15 species of *Lauraceae* family from NCBI were aligned using MAFFT v.7 (Katoh and Standley 2013). The auto algorithm in the MAFFT alignment software was used to align the 18 complete genome sequences and the G-INS-i algorithm was used to align the partial complex sequences. The maximum likelihood (ML) bootstrap analysis was conducted using RAxML (Stamatakis 2006); bootstrap probability values were calculated from 1000 replicates. *Chimonanthus nitens* (MH377058) and *Chimonanthus praecox* (MH377057) were served as the out-group.

The complete *L. garrettii* plastid genome is a circular DNA molecule with the length of 154,011 bp, contains a large single-copy region (LSC) of 93,697 bp and a small single-copy region (SSC) of 18,826 bp, which were separated by a pair of inverted repeats (IR) regions of 20,744 bp. The overall GC content of the whole genome is 39.2% and the corresponding values of the LSC, SSC, and IR regions are 38.0, 34.0, and 44.3%, respectively. The plastid genome contained 127 genes, including 82 protein-coding genes, 8 ribosomal RNA genes, and 36 transfer RNA genes. Phylogenetic analysis showed that *L. garrettii* and *Parasassafras confertiflorum* clustered in a unique clade in *Lauraceae* family (Figure 1). The determination of the complete plastid genome sequences provided new molecular data to illuminate the *Lauraceae* family evolution.

**Figure 1.**
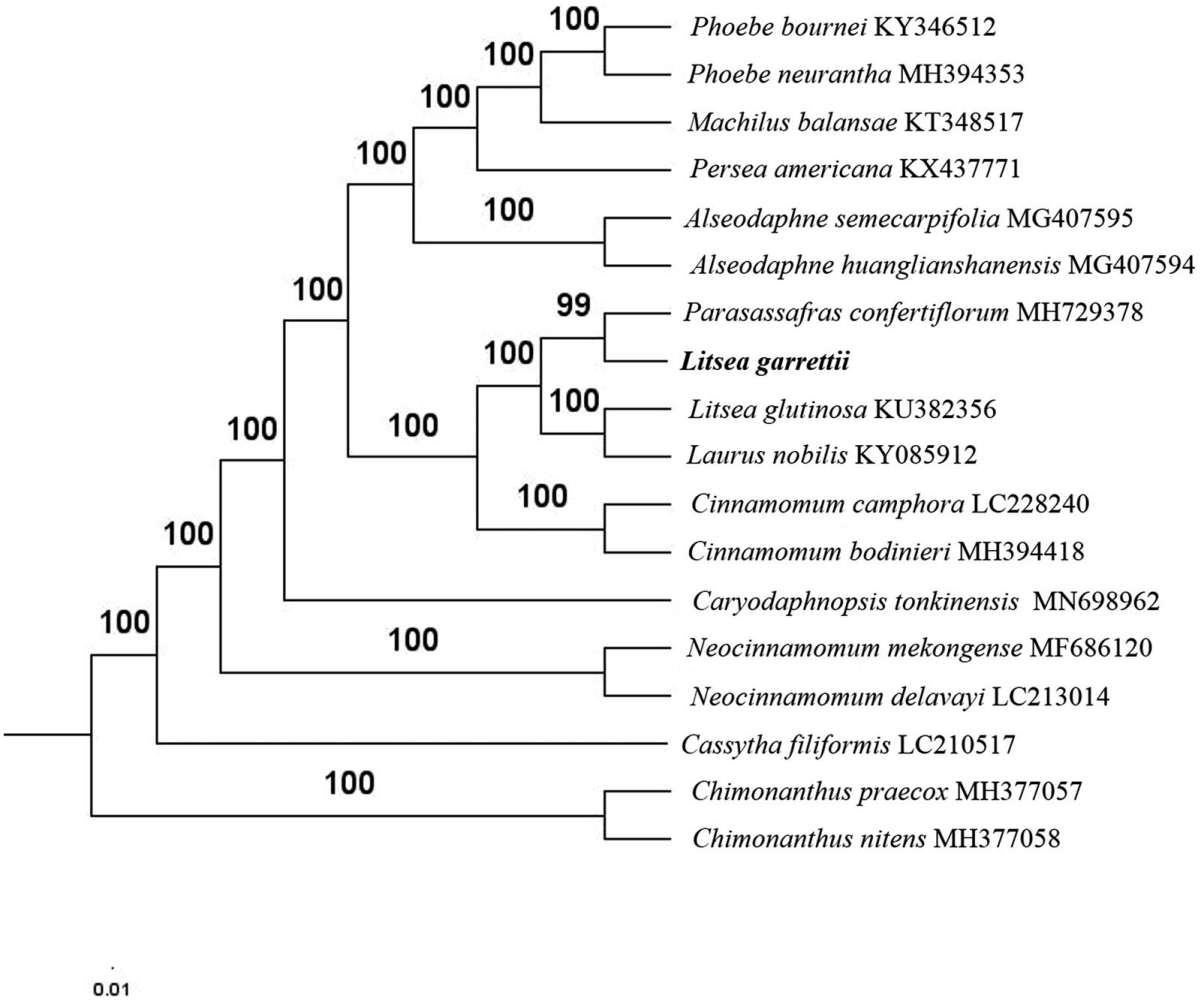
The maximum-likelihood tree based on the 16 chloroplast genomes of *Lauraceae* family. The bootstrap value based on 1000 replicates is shown on each node.
